# Validation of self-reported measures of nutritional status: a study based on the PNS 2019

**DOI:** 10.11606/s1518-8787.2024058005505

**Published:** 2024-07-10

**Authors:** Renatha Celiana da Silva Brito, Angelo Giuseppe Roncalli da Costa Oliveira

**Affiliations:** I Universidade Federal do Rio Grande do Norte Programa de Pós-Graduação em Saúde Coletiva Natal RN Brasil Universidade Federal do Rio Grande do Norte. Programa de Pós-Graduação em Saúde Coletiva. Natal, RN, Brasil; II Universidade Federal do Rio Grande do Norte Departamento de Odontologia Natal RN Brasil Universidade Federal do Rio Grande do Norte. Departamento de Odontologia. Natal, RN, Brasil

**Keywords:** Epidemiology, Validation Study, Nutritional Status, Public Health

## Abstract

**OBJECTIVE:**

To analyze the validity of self-reported anthropometric measurements (weight and height) for classifying the nutritional status of Brazilian adults and elderly people using data from the 2019 National Health Survey (PNS).

**METHODS:**

The PNS sample is made up of permanent private households from all of Brazil’s federative units and this is a cross-sectional study in which 6,571 records were identified with measured and reported data, with no missing data for one variable being identified when in the presence of another. Validation was carried out with 6,381 data after removing atypical data. The variables used for stratification were: gender, age, race/color, schooling, and income, and the weighted Kappa Coefficient and the Intraclass Correlation Coefficient (ICC) were used to analyze agreement between the nutritional status categories. Accuracy was analyzed based on sensitivity, specificity, positive predictive value (PPV) and negative predictive value (NPV). For construct validity, a Poisson regression was performed for each outcome (measured and self-reported), with the independent variables “gender”, “color/race”, “schooling”, and “family income”.

**RESULTS:**

All the analyses showed positive results for validation. There was greater reproducibility among adults (18 to 59 years old) compared to the elderly and among men compared to women.

**CONCLUSION:**

This validation indicates a concrete possibility of carrying out an association of observational studies using reported nutritional status as the outcome variable, as an efficient strategy which could minimize the operational difficulties often encountered.

## INTRODUCTION

An individual’s nutritional status is characterized by the relationship between their intake and expenditure of nutrients which, in turn, is determined by the body’s need and ability to digest, absorb, and use the nutrients ingested^1^. This nutritional status is identified using anthropometric variables such as height and body mass, which are consequently classified according to their positive or negative relationship with health. Anthropometry is a simple method to use in clinical practice but it is very logistical when it comes to population surveys^2^.

Overweight is an important public health problem, given its rampant and progressive increase in recent decades worldwide, characterized by multifactorial causes, which encompasses a complex interaction between genetic predispositions, environmental factors and lifestyle^1,2^. In Brazil, in 2018, 55.7% of the adult population was overweight, of which 19.8% was classified of obesity^3^. More recent studies show that out of a total of 12 million adults followed up in Primary Health Care (PHC), 8 million (63.0%) were overweight, of which 2.6 million (28.5%) were classified of obesity^4^.

Monitoring the nutritional status of the Brazilian population is essential not only to understand the population profile, but also to plan and evaluate public policies at different levels of health care. For this reason, surveys using anthropometry are carried out. This data makes it possible to find out about trends in overweight and obesity in different geographical areas, with different population groups, and to identify the main associated factors^5^.

However, these surveys require high costs and logistics for them to be effective, with anthropometric assessment being one of the modules with the greatest limitations, such as the acquisition of suitable instruments, difficulties in transporting the material, standardization, and poor mastery of measurement techniques, training, increased fieldwork time, among others^6^.

In this sense, several surveys have used the strategy of self-reporting weight and height - direct measurements used to calculate the Body Mass Index (BMI) and, consequently, classification of nutritional status - to minimize the difficulties encountered, since they promote resource savings and simplify fieldwork^9,10^. In Brazil, for example, the Surveillance System of Risk and Protective Factors for Chronic Diseases by Telephone Survey for adults in Brazil (Vigitel) program has been using the self-report technique since 2006 and, in its 2019 edition, showed that obesity among Brazilians over 18 increased by 72.0% between 2006 and 2019, from 11.8% to 20.3%^4,11^.

The National Health Survey (Pesquisa Nacional de Saúde - PNS), a large survey with a sample from all Brazilian regions, has already been carried out in two editions (2013 and 2019). In addition to using measured weight and height, it has also used self-reported measures and its main objective is to produce data on the health and lifestyles of the population, in addition to providing knowledge about access to and use of health services by users^12^.

Therefore, the aim of this study was to analyze the validity of self-reported anthropometric measurements (weight and height) for classifying the nutritional status of Brazilian adults and elderly people based on data from the 2019 PNS.

## METHODS

The PNS sample is made up of residents of permanent private households (built exclusively for housing purposes) in Brazil, in rural and urban areas, except for special census tracts (lodgings, long-stay institutions for the elderly, camps, among others) or those with little housing.

Sampling was done by conglomerates in three stages, with stratification of the Primary Sampling Units (PSU). For all stages, the PSUs were selected by simple random sampling, and this edition of the PNS was approved by the National Committee of Ethics in Research (Conep) under opinion No. 3.529.376, with the Brazilian Institute of Geography and Statistics (IBGE) being responsible for the fieldwork. All the individuals selected for the study gave their consent for the questionnaire to be administered and for their anthropometric measurements to be taken^12^.

In 2019, IBGE interviewers conducted 94,114 interviews with male and female residents aged 15 and over in all Brazilian state capitals. To this end, self-reported and measured anthropometric data were collected from 87,678 and 6,571 individuals, respectively, aged 18 and older (excluding 736 data from individuals aged between 15 and 17 for analysis in this study). Self-reported anthropometric data was not collected from women who reported being pregnant at the time of the interview.

Self-reported data was collected in the Lifestyle Module using the questions: “Do you know your weight?” and “Do you know your height?”, the answers to which were recorded in kilograms (kg) and centimeters (cm). The measurements were taken with a defined sub-sample allocated proportionally to the strata, with at least two PSUs per stratum.

The measurements were taken using the same procedures used in the 2008-2009 Consumer Expenditure Survey (POF) conducted by the Ministry of Health in partnership with the IBGE^13^, by teams trained by the Oswaldo Cruz Foundation (Fiocruz) and the Laboratory of Nutritional Assessment of Populations (Lanpop) at the University of São Paulo’s School of Public Health (FSP/USP), using portable stadiometers and digital scales.

For both measured and self-reported data, nutritional status was classified based on BMI, obtained from the ratio between the individual’s weight and height squared (weight/height^2^), considering the reference values recommended by the Ministry of Health^3^ for adults (18 to 59 years): ≤ 18.4 kg/m^2^ for underweight, between 18.5 and 24.9 kg/m^2^ for eutrophy and ≥ 25 kg/m^2^ for overweight^14^; and for the elderly (aged 60 or over): ≤ 21.9 kg/m^2^ for underweight, between 22 and 26.9 kg/m^2^ for eutrophic and ≥ 27 kg/m^2^ for overweight^15^. In the analyses, BMI data was used as a categorical variable, based on its classification.

The variables used for stratification were: gender (male/female); age - classified as “non-elderly” for those aged between 18 and 59 and “elderly” for those aged 60 or older; race or color - with a dichotomous classification as “white” and “non-white” (grouping those who declared themselves as “black”, “yellow”, “brown”, or “indigenous”); schooling - dichotomous classification of yes/no for the question “Can you read and write?”; income - classified as “≤ 1 minimum wage” and “> 1 minimum wage”, considering the minimum wage for 2019, equivalent to R$998.00.

Initially, to check the consistency between the measured and self-reported data, an analysis of atypical data (outliers) was carried out to identify possible recording errors using the multivariate detection technique, calculating the Mahalanobis D^2^distance.

Next, to analyze the reliability between the nutritional status categories, the Chi^2^ test was used, along with the Phi coefficient (which has a result ranging from -1 to 1, where zero indicates that there is no relationship between the variables, while values close to the extremes -1 and 1 indicate a strong correlation), the weighted and unweighted Kappa Coefficient, the Pearson correlation coefficient and the intraclass correlation coefficient (ICC) were used, adopting the Landis and Koch criterion^16^ , considering the following levels of reliability: none (less than zero); discrete (0 to 0.20); regular (0.21 to 0.40); moderate (0.41 to 0.60); substantial (0.61 to 0.80); and perfect (0.81 to 1.00).

Accuracy was analyzed based on the values of sensitivity, specificity, positive predictive value (PPV) and negative predictive value (NPV), using the measurements taken to classify nutritional status (BMI) as the gold standard.

For construct validity, a Poisson regression was performed for each outcome (measured and self-reported), with the independent variables “gender”, “color/race”, “schooling”, and “family income”. Stata software, version 14.0, was used for all statistical analyses, considering a 95% confidence interval (CI) and a 5% significance level (p). The prevalence ratio (PR) measure was used for the association tests.

## RESULTS

### Data Consistency Analysis

A total of 6,571 records were identified with measured and reported weight and height data and, consequently, estimated BMI, and no data was lost for one variable when another was present. This sample has the following characteristics: in terms of gender, 50.2% are men (n = 3,298) and 49.8% are women (n = 3,273), with an average age of 48. As for schooling, 87.6% answered “yes” to “can read and write” (n = 5,756) and 12.4% “no” (n = 815); as for race/color, 60.2% self-declared as black, yellow, brown, or indigenous (n = 3,954) and 39.8% self-declared as white (n = 2,617). As for income, 59.4% of individuals reported having a family income of one minimum wage or less (n = 3,901).

An analysis of atypical data (outliers) was then carried out in order to identify possible recording errors. In an initial analysis, considering the normalized values, some values above three standard deviations were observed, especially at the upper limit. An analysis of atypical data was then carried out using the multivariate detection technique, calculating the Mahalanobis D^2^ distance and its respective probability, in accordance with the approach recommended by Hair et al.^17^, and 190 atypical data (2.9%) were identified, which can be seen in the Figure. Thus, 6,381 records of measured BMI and reported BMI were included in the validation analysis.

**Figure:**
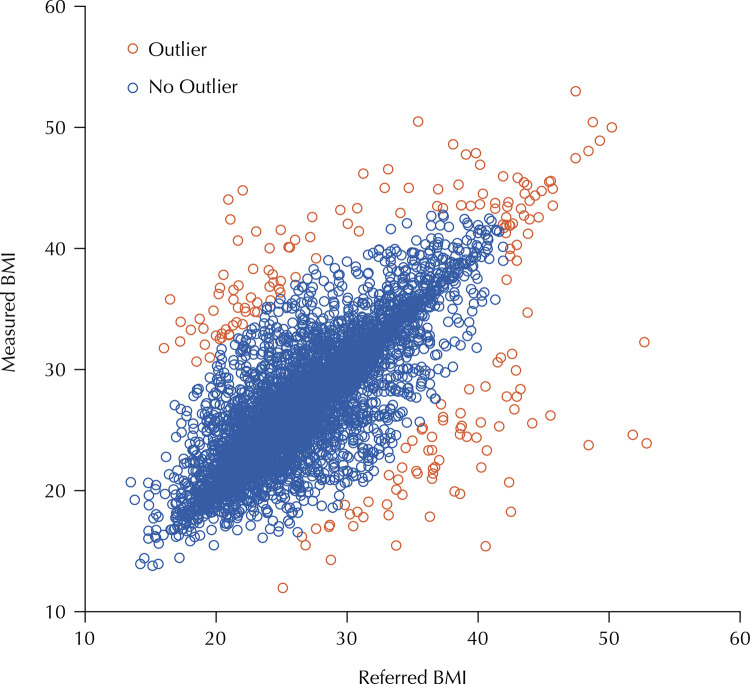
Identification of atypical data for Body Mass Index (BMI) using the multivariate detection technique.

Source: Authors’ analysis of data from the National Health Survey (PNS), 2019.

### Reliability Analysis - BMI Variable in Quantitative Form

Reliability was assessed by calculating Pearson’s correlation coefficient and ICC based on BMI in quantitative form. A simple linear regression was also carried out, with measured BMI as the dependent variable and reported BMI as the independent variable.

Pearson’s correlation coefficient was 0.826 (p < 0.001), indicating a strong correlation. However, this coefficient has a natural bias, which is that it shows the correlation without considering the agreement between the values. In this sense, the ICC was also calculated, with a value of 0.904 (p < 0.001; 95%CI 0.899–0.909), which is considered perfect.

The linear regression showed an R^2^ of 0.682, indicating that 68.2% of the variation in measured BMI can be credited to reported BMI. The model proved to be significant by the Analysis of Variance (p < 0.001) and the resulting final equation was: measured BMI=3.96+0.864× reported BMI . The evaluation of the model’s predictive power showed a variation in the standardized residuals between -3.9 and 4.0, with a normal distribution and a Durbin-Watson value of 1.32, indicating independence between the residuals.

### Reliability Analysis - BMI Variable in Categorized Form

There was an association between the categories of reported BMI and measured BMI, using the Chi^2^ test equal to 4.711 and a significance value of p < 0.001, considering the limits for classifying BMI for adults and the elderly, characterized as “underweight”, “eutrophic”, and “overweight”.

In addition to observing significance, the Phi coefficient was calculated and found to be 0.859 (p < 0.001), indicating a strong association. The unweighted Kappa was 0.637 (p < 0.001; 95%CI: 0.619–0.654). However, considering that this is an ordinal variable, the weighted Kappa can better express agreement, and a value of 0.664 (p < 0.001; 95%CI: 0.648–0.681) was found, indicating good agreement for the nutritional status categories. When the weighted Kappa was assessed in a stratified manner (Tables 1 and 2), we saw that there was good reliability for both gender (0.681 for men and 0.646 for women) and age (0.684 for the non-elderly and 0.587 for the elderly).

**Table 1:** Bivariate analysis by gender, following the categories of measured body mass index and reported body mass index. Brazil, 2023.

Category	Referred BMI classification	Classification of measured BMI			Total	Weighted Kappa	95%CI	p-value
	
Sex		Underweight	Eutrophic	Overweight				
Men	Underweight	104	72	6	182	0.681	0.658–0.704	0.001
	Eutrophic	79	1,067	239	1,385			
	Overweight	11	207	1,468	1,686			
	Total	194	1,346	1,713	3,253			
Women	Underweight	103	66	11	180	0.646	0.622–0.671	0.001
	Eutrophic	56	901	346	1,303			
	Overweight	8	158	1,479	1,645			
	Total	167	1,125	1,836	3,128			
Total	Underweight	207	138	17	362			
	Eutrophic	135	1,968	585	2,688			
	Overweight	19	365	2,947	3,331			
	Total	361	2,471	3,549	6,381			

BMI: body mass index; CI: confidence interval.

Source: Authors’ analysis of data from the National Health Survey (PNS) 2019

**Table 2:** Bivariate analysis by age, following the categories of measured body mass index and reported body mass index. Brazil, 2023.

Category	Referred BMI classification	Classification of measured BMI			Total	Weighted Kappa	95%CI	p-value
	
Age		Underweight	Eutrophic	Overweight				
Not elderly	Underweight	28	19	2	49	0.684	0.664–0.704	0.001
	Eutrophic	18	678	228	924			
	Overweight	1	108	1,204	1,313			
	Total	47	805	1,434	2,286			
Elderly	Underweight	75	47	9	131	0.587	0.555–0.620	0.001
	Eutrophic	38	223	118	379			
	Overweight	7	50	275	332			
	Total	120	320	402	842			
Total	Underweight	103	66	11	180			
	Eutrophic	56	901	346	1,303			
	Overweight	8	158	1,479	1,645			
	Total	167	1,125	1,836	3,128			

BMI: body mass index; CI: confidence interval.

Source: Authors’ analysis of data from the National Health Survey (PNS) 2019

### Categorized BMI Analysis - Criterion Validity (Accuracy)

Accuracy statistics were also calculated, considering measured BMI as the gold standard. For this purpose, overweight was considered as the outcome. The parameters were also calculated stratified by sex (men and women) and age (non-elderly and elderly). The results are shown in Table 3.

**Table 3:** Parameters found for the analysis of accuracy between measured body mass index and reported body mass index according to the subgroups studied and for the total sample. Brazil, 2023.

Variable	Sensitivity	Specificity	PPV	NPV	Diagnostic accuracy

	% (95%CI)	% (95%CI)	% (95%CI)	% (95%CI)	% (95%CI)
Men	85.7 (84.0–87.3)	85.8 (84.0–87.5)	87.1 (85.4–88.6)	84.4 (82.5–86.1)	85.8 (84.5–86.9)
Women	80.6 (78.7–82.3)	87.2 (85.2–88.9)	89.9 (88.4–91.3)	75.9 (73.7–78.0)	83.3 (81.9–84.6)
Not elderly	85.8 (84.5–87.0)	85.0 (83.3–86.6)	89.7 (88.5–90.8)	79.6 (77.8–81.4)	85.5 (84.4–86.5)
Elderly	72.1 (68.7–75.3)	89.2 (87.1–91.0)	82.9 (79.8–85.7)	81.4 (79.0–83.7)	82.0 (80.1–83.8)
Total sample	83.0 (81.8–84.2)	86.4 (85.1–87.6)	88.5 (87.3–89.5)	80.3 (78.8–81.6)	84.6 (83.6–85.4)

PPV: positive predictive value; NPV: negative predictive value; CI: confidence interval.

Source: Authors’ analysis of data from the National Health Survey (PNS) 2019

Sensitivity, the ability of overweight individuals to report their weight and height data correctly, was high at 83.0% (95%CI between 81.8% and 84.2%), while specificity, the ability of non-overweight individuals to report their data correctly, was also high at 86.4% (95%CI between 85.1% and 87.7%).). When stratified by sex and age, we see that overweight women and elderly people (≥ 60 years) have lower sensitivity for self-reporting their nutritional status, 80.6 and 72.1%, respectively.

### Analysis of Categorized BMI - Construct Validity

Finally, categorized BMI was analyzed according to some predictor variables that are commonly associated with nutritional status, such as: gender, race/color, schooling and income^8,18^. The Poisson regression analysis for reported BMI and measured BMI (Table 4) showed that the only variable that differed in the two models was gender (which was significant for measured BMI and not for reported BMI). This reinforces the need to always stratify by sex when using reported BMI, corroborating the previous results.

**Table 4:** Poisson regression analysis for reported body mass index and measured body mass index. Brazil, 2023.

BMI	Variable	Adjusted PR	95%CI	p-value
Referred BMI	Sex	1.022	0.974–1.072	0.367
	Color/race	1.031	0.982–1.083	0.217
	Education	0.781	0.715–0.854	0.001
	Family income	0.94	0.895–0.988	0.014
Measured BMI	Sex	1.122	1.073–1.174	0.001
	Color/race	1.002	0.958–1.048	0.925
	Education	0.812	0.748–0.881	0.001
	Family income	0.948	0.906–0.993	0.022

BMI: body mass index; PR: prevalence ratio; CI: confidence interval.

Source: Authors’ analysis of data from the National Health Survey (PNS) 2019.

## DISCUSSION

Through the series of statistical analyses carried out to validate the measured and reported weight and height data (and consequent estimation of BMI), this study showed that the self-reference technique can be used in population surveys and is an efficient strategy to minimize the difficulties often encountered (especially in terms of logistics and time) in directly measuring these anthropometric measurements.

There was greater reproducibility among non-elderly adults (18 to 59 years old) compared to elderly people and among men compared to women. This conclusion is reinforced when we see that, among the socioeconomic and demographic variables analyzed, the only one that has a difference in all the statistical models is gender, corroborating some similar studies^8,18^.

Sensitivity among women was 80.6%, while among men it was 87.0%, meaning that among overweight women and men, women were able to report their data less accurately. On the other hand, when they were not overweight, women had a higher specificity (87.2%), in line with other studies which have shown that people who are not overweight are able to report their measurements more accurately^7^.

The results of this study on women reinforce the hypothesis that, while they are more concerned about monitoring their measurements and health status^21^, in general, when compared to men, they also have a greater tendency to underestimate their weight and overestimate their height^6,20,22^, which can lead to important fluctuations in the definition of their nutritional status. This can be explained by the fact that this group often has higher levels of body dissatisfaction, triggered by high social pressure to achieve certain standards of beauty^23,24^. In general, when individuals feel more satisfied with their own body image or are closer to the body weight of their peers, they are less likely to misreport their weight.^21^

Elderly people showed lower sensitivity (72.1%) when compared to non-elderly adults (85.0%), i.e. overweight elderly people are less accurate in reporting their anthropometric data, while elderly people who are not overweight (specificity of 89.2%) are able to report more accurately, which corroborates other similar studies^20,25^. This may be associated with the physiological process of ageing itself^26^, which leads to a decrease in height over the years, as well as a progressive reduction in muscle mass and/or increase in adipose tissue due to a decrease in metabolic rate, impacting on changes in body weight, often not perceived by the elderly^27,28^.

In addition, this is a group that generally does not check its weight and height regularly, thus leading to more inaccurate and outdated information, which may also be associated with memory bias, commonly present in this public, corroborating these results with other studies that observed an underestimation of weight and overestimation of height in the elderly^18,20^.

This validation of the PNS is very important, given the wide use of this database in cross-sectional and longitudinal studies to assess morbidity, the functioning of health care and the lifestyle of the Brazilian population, in different areas of knowledge due to the wealth of variables, large sample size and representativeness of all the federative units in Brazil.

Furthermore, although we found good agreement for the categories of nutritional status, showing that the PNS 2019 can be used to estimate the prevalence of overweight in this population, other population-based studies, different from this one, found values of underestimation^29,30^ and overestimation for overweight and obesity^18^.

To validate the construct, we used some predictors that the literature commonly shows to be associated with nutritional status (gender, race/color, schooling, and income), which reinforced the legitimacy of using self-reported data in association studies, due to its similarity to measured data. Therefore, although caution is recommended when using self-reported data for prevalence studies of nutritional status for overweight, especially with the elderly and women, the results of this study indicate that there is a real possibility of carrying out association studies using self-reported nutritional status as the outcome variable.

This becomes even more relevant when it comes to large population surveys, where there is a high cost and logistical demand for fieldwork, based on the need for training, the acquisition of suitable materials in large quantities, difficulties with transportation and the long duration of the collections. The use of self-reported anthropometric data increases the feasibility of observational studies, pointing to the importance of good planning of the research design, considering its central objective and the characteristics of the target population, especially sociocultural, demographic and economic.
