# Supplementary material for: Validation of self-reported measures of nutritional status: a study based on the PNS 2019
Source: Rev Saude Publica. 2024 Jul 10;58:28. doi: 10.11606/s1518-8787.2024058005505 (PMC11319040; doi:10.11606/s1518-8787.2024058005505)
Supplement: Translation

# Translation: Validação de medidas autorreferidas para estado nutricional: estudo a partir da PNS 2019

**Authors:** Renatha Celiana da Silva Brito, Angelo Giuseppe Roncalli da Costa Oliveira

**Affiliations:** I Brasil Universidade Federal do Rio Grande do Norte. Programa de Pós-Graduação em Saúde Coletiva. Natal, RN, Brasil; II Brasil Universidade Federal do Rio Grande do Norte. Departamento de Odontologia. Natal, RN, Brasil

**Keywords:** Epidemiologia, Estudo de Validação, Estado Nutricional, Saúde Pública

## Abstract

**OBJETIVO:**

Analisar a validade da autorreferência de medidas antropométricas (peso e altura) para a classificação do estado nutricional de adultos e idosos brasileiros a partir dos dados da Pesquisa Nacional de Saúde (PNS), edição de 2019.

**MÉTODOS:**

A amostra da PNS é constituída por domicílios particulares permanentes de todas as unidades federativas do Brasil e este é um recorte transversal no qual foram identificados 6.571 registros com dados aferidos e referidos, não sendo identificados dados perdidos para uma variável quando na presença de outra. A validação foi realizada com 6.381 dados após a retirada de dados atípicos. As variáveis utilizadas para estratificação foram: sexo, idade, raça/cor, escolaridade e renda e, para analisar a concordância entre as categorias do estado nutricional, foram utilizados o Coeficiente Kappa ponderado e o Coeficiente de Correlação Intraclasse (CCI). A acurácia foi analisada com base nos valores de sensibilidade, especificidade, valor preditivo positivo (VPP) e valor preditivo negativo (VPN). E para a validade de constructo, foi realizada uma regressão de Poisson para cada desfecho (aferido e autorreferido), com as variáveis independentes “sexo”, “cor/raça”, “escolaridade” e “renda familiar”.

**RESULTADOS:**

Todas as análises mostraram resultados positivos para a validação. Houve uma maior reprodutibilidade entre adultos (18 a 59 anos) quando comparados às pessoas idosas e entre homens quando comparados às mulheres.

**CONCLUSÃO:**

Esta validação indica uma possibilidade concreta de realizar estudos observacionais de associação tendo como variável de desfecho o estado nutricional referido, como uma estratégia eficiente, podendo minimizar as dificuldades operacionais frequentemente encontradas.

## INTRODUÇÃO

O estado nutricional de um indivíduo é caracterizado pela relação entre sua ingestão e gasto de nutrientes que, por sua vez, é determinada pela necessidade e capacidade que o corpo tem de digerir, absorver e utilizar os nutrientes ingeridos^1^. Este estado nutricional é identificado a partir de variáveis antropométricas de estatura e massa corporal que, consequentemente, é classificado quanto a sua relação positiva ou negativa com a saúde, sendo a antropometria um método de simples utilização na prática clínica, porém de grande logística quando se trata de inquéritos populacionais^2^.

O excesso de peso é um importante problema de Saúde Pública, tendo em vista seu aumento desenfreado e progressivo nas últimas décadas de forma mundial, caracterizado por causas multifatoriais, que engloba uma interação complexa entre predisposições genéticas, fatores ambientais e estilo de vida^1,2^. No Brasil, em 2018, 55,7% da população adulta brasileira possuía excesso de peso, dos quais 19,8% estavam classificadas com obesidade^3^. Já estudos mais recentes mostram que de um total de 12 milhões de adultos acompanhados na Atenção Primária à Saúde (APS), 8 milhões (63,0%) apresentaram excesso de peso, dos quais 2,6 milhões (28,5%) apresentaram o estado nutricional de obesidade^4^.

O monitoramento do estado nutricional da população brasileira é essencial para, além de conhecer o perfil populacional, planejar e avaliar as políticas públicas nos diferentes níveis de atenção à saúde. Por isso, são realizados inquéritos que utilizam a antropometria como objeto de coleta. Esses dados permitem conhecer as tendências de sobrepeso e obesidade em diferentes áreas geográficas, com grupos populacionais distintos, e identificar os principais fatores associados^5^.

No entanto, essas pesquisas demandam alto custo e grande logística para sua eficácia, sendo a avaliação antropométrica um dos módulos com maiores limitações, como a aquisição de instrumentos adequados, dificuldades de transporte do material, padronização e baixo domínio das técnicas de aferição, treinamentos, aumento do tempo de trabalho de campo, entre outros^6^.

Neste sentido, vários inquéritos têm utilizado a estratégia de autorreferência de peso e altura – medidas diretas utilizadas para o cálculo do Índice de Massa Corporal (IMC) e, consequentemente, classificação de estado nutricional – para minimizar as dificuldades encontradas, uma vez que promovem a economia de recursos e simplifica o trabalho de campo^9,10^. No Brasil, por exemplo, o programa de Vigilância dos Fatores de Risco e Proteção para Doenças Crônicas por Inquérito Telefônico (Vigitel) vem utilizando a técnica de autorrelato desde 2006 e, em sua edição de 2019, mostrou que a obesidade entre brasileiros maiores de 18 anos aumentou 72,0% entre 2006 e 2019, passando de 11,8% para 20,3%^4,11^.

A Pesquisa Nacional de Saúde (PNS), grande inquérito com amostra de todas as regiões brasileiras, já realizada em duas edições (2013 e 2019), além de utilizar medidas de peso e altura aferidas, também tem utilizado medidas autorreferidas e tem como principal objetivo produzir dados sobre a saúde e os estilos de vida da população, além de trazer conhecimento sobre o acesso e o uso dos serviços de saúde pelos usuários^12^.

Sendo assim, o presente estudo teve o objetivo de analisar a validade da autorreferência de medidas antropométricas (peso e altura) para a classificação do estado nutricional de adultos e idosos brasileiros a partir de dados da PNS, edição de 2019.

## MÉTODOS

A amostra da PNS é constituída por moradores dos domicílios particulares permanentes (construídos com a finalidade exclusiva de habitação) do Brasil, da zona rural e zona urbana, com exceção dos setores censitários especiais (alojamentos, instituições de longa permanência para idosos, acampamentos, dentre outros) ou com pouca habitação.

A amostragem deu-se por conglomerados em três estágios, com estratificação das Unidades Primárias de Amostragem (UPA). Para todas as etapas, a seleção das UPA foi realizada por amostragem aleatória simples, e esta edição da PNS foi aprovada pela Comissão Nacional de Ética em Pesquisa (Conep) sob o parecer nº 3.529.376, tendo sido o Instituto Brasileiro de Geografia e Estatística (IBGE) responsável pelo trabalho de campo. Todos os indivíduos selecionados para a pesquisa forneceram consentimento para a aplicação do questionário e para a aferição de suas medidas antropométricas^12^.

Em 2019, os entrevistadores do IBGE realizaram 94.114 entrevistas com moradores do sexo masculino e feminino, a partir dos 15 anos, distribuídos em todas as capitais brasileiras. Para tanto, foram coletados dados antropométricos autorreferidos e aferidos de 87.678 e 6.571 indivíduos, respectivamente, com idade igual ou superior a 18 anos (excluindo 736 dados de indivíduos entre 15 e 17 anos para análise do presente estudo). Não foram coletados dados antropométricos aferidos e referidos de mulheres que relataram estar gestantes no momento da entrevista.

Os dados autorreferidos foram coletados no Módulo de Estilo de Vida por meio das perguntas: “O(a) senhor(a) sabe o seu peso?” e “O(a) senhor(a) sabe a sua altura?”, cujas respostas foram registradas em quilogramas (kg) e em centímetros (cm). A aferição das medidas aconteceu com uma subamostra definida e alocada proporcionalmente nos estratos com, no mínimo, duas UPA por estrato.

Para a aferição das medidas foram utilizados os mesmos procedimentos da Pesquisa de Orçamento Familiar (POF) 2008–2009, do Ministério da Saúde em parceria com o IBGE^13^, por equipes treinadas pela Fundação Oswaldo Cruz (Fiocruz) e pelo Laboratório de Avaliação Nutricional de Populações (Lanpop) da Faculdade de Saúde Pública da Universidade de São Paulo (FSP/USP), utilizando estadiômetros portáteis e balanças digitais.

Tanto para os dados aferidos quanto para os dados autorreferidos, a classificação do estado nutricional foi feita a partir do IMC, obtido pela razão entre o peso e a altura ao quadrado do indivíduo (peso/altura^2^), considerando os valores de referência recomendados pelo Ministério da Saúde^3^ para adultos (18 a 59 anos): ≤ 18,4 kg/m^2^ para baixo peso, entre 18,5 e 24,9 kg/m^2^ para eutrofia e ≥ 25 kg/m^2^ para excesso de peso^14^; e para idosos (maior ou igual a 60 anos): ≤ 21,9 kg/m^2^ para baixo peso, entre 22 e 26,9 kg/m^2^ para eutrofia e ≥ 27 kg/m^2^ para excesso de peso^15^. Nas análises, portanto, os dados de IMC foram utilizados enquanto variável categórica, a partir de sua classificação.

As variáveis utilizadas para estratificação foram: sexo (homem/mulher); idade – classificada em “não idoso” para aquele com idade entre 18 e 59 anos e em “idoso” para os com 60 anos ou mais; raça ou cor – com a classificação dicotômica em “branco” e “não branco” (agrupamento dos que se autodeclararam como “preto”, “amarelo”, “pardo” ou “indígena”); escolaridade – classificação dicotômica de sim/não para a pergunta “O senhor(a) sabe ler e escrever?”; renda – classificada em “≤ 1 salário mínimo” e “> 1 salário mínimo”, considerando o salário mínimo referente ao ano de 2019, equivalente a R$ 998,00.

Inicialmente, para verificar a consistência entre os dados aferidos e autorreferidos, foi realizada uma análise de dados atípicos (*outliers*), para identificar possíveis erros de registros a partir da técnica de detecção multivariada, com o cálculo da distância D^2^ de Mahalanobis.

Na sequência, para analisar a confiabilidade entre as categorias do estado nutricional, foram utilizados o teste de Qui^2^ juntamente com o coeficiente Phi (que possui um resultado que vai de -1 a 1, no qual o zero indica que não há relação entre as variáveis, enquanto os valores próximos aos extremos -1 e 1 indicam correlação forte), o Coeficiente Kappa ponderado e não ponderado, o coeficiente de correlação de Pearson e o coeficiente de correlação intraclasse (CCI), adotando-se o critério de Landis e Koch^16^, considerando os seguintes níveis de confiabilidade: nenhuma (menor que zero); discreta (0 a 0,20); regular (0,21 a 0,40); moderada (0,41 a 0,60); substancial (0,61 a 0,80); e perfeita (0,81 a 1,00).

A acurácia foi analisada com base nos valores de sensibilidade, especificidade, valor preditivo positivo (VPP) e valor preditivo negativo (VPN), tomando como padrão-ouro as medidas aferidas para a classificação do estado nutricional (IMC).

Para a validade de constructo, foi realizada uma regressão de Poisson para cada desfecho (aferido e autorreferido), com as variáveis independentes “sexo”, “cor/raça”, “escolaridade” e “renda familiar”. Para todas as análises estatísticas, foi utilizado o *software* Stata, versão 14.0, considerando o intervalo de confiança (IC) de 95% e nível de significância (p) de 5%. Para os testes de associação, foi utilizada a medida de razão de prevalência (RP).

## RESULTADOS

### Análise de Consistência dos Dados

Foram identificados 6.571 registros com dados aferidos e referidos de peso e altura e, consequentemente, estimativa de IMC, não sendo identificados dados perdidos para uma variável quando na presença de outra. Essa amostra tem as seguintes características: quanto ao sexo, 50,2% são homens (n = 3.298) e 49,8% são mulheres (n = 3.273), com idade média de 48 anos. Quanto à escolaridade, 87,6% responderam “sim” para “sabe ler e escrever” (n = 5.756) e 12,4% “não” (n = 815); quanto à raça/cor, 60,2% se autodeclaram como pretos, amarelos, pardos ou indígenas (n = 3.954) e 39,8% se autodeclaram brancos (n = 2.617). Quanto à renda, 59,4% dos indivíduos relataram ter renda familiar igual ou menor a 1 salário-mínimo (n = 3.901).

Foi então realizada a análise de dados atípicos (*outliers*) no sentido de identificar possíveis erros de registro. Em uma análise inicial, considerando os valores normalizados, foram observados alguns valores acima de três desvios-padrões, principalmente no limite superior. Desse modo, foi realizada uma análise de dados atípicos a partir da técnica de detecção multivariada, com o cálculo da distância D^2^ de Mahalanobis e sua respectiva probabilidade, de acordo com a abordagem preconizada por Hair et al^17^, sendo identificados 190 dados atípicos (2,9%), os quais podem ser observados na Figura. Assim, foram incluídos na análise de validação 6.381 registros de IMC aferido e de IMC referido.

**Figura:** Identificação dos dados atípicos para o Índice de Massa Corporal (IMC) pela técnica de detecção multivariada.

Fonte: Análise dos autores sobre dados da Pesquisa Nacional de Saúde (PNS), 2019.

### Análise de Confiabilidade – Variável IMC na Forma Quantitativa

A avaliação da confiabilidade, tendo como base o IMC na forma quantitativa, se deu a partir do cálculo do coeficiente de correlação de Pearson e do CCI. Além disso, foi realizada uma regressão linear simples, tendo o IMC aferido como variável dependente e o IMC referido como variável independente.

O Coeficiente de Correlação de Pearson foi igual a 0,826 (p < 0,001) indicando uma correlação forte. Porém, este coeficiente tem um viés natural que é o fato de mostrar a correlação sem considerar a concordância entre os valores. Neste sentido, foi calculado também o CCI, cujo valor foi de 0,904 (p < 0,001; IC95% 0,899–0,909), considerado perfeito.

A regressão linear mostrou um R^2^ de 0,682, indicando que 68,2% da variação do IMC aferido pode ser creditada ao IMC referido. O modelo revelou-se significativo pela Análise de Variância (p < 0,001) e a equação final resultante foi:  IMC aferido =3.96+0.864× IMC referido . A avaliação do poder preditivo do modelo mostrou uma variação dos resíduos padronizados entre -3,9 e 4,0 e com distribuição normal e o valor do Durbin-Watson foi de 1,32, indicando independência entre os resíduos.

### Análise de Confiabilidade – Variável IMC na Forma Categorizada

Houve associação entre as categorias do IMC referido e do IMC aferido, através do teste de Qui^2^ igual a 4.711 e valor de significância de p < 0,001, considerando os limites para a classificação do IMC, para adultos e idosos, caracterizados em “baixo peso”, “eutrofia” e “excesso de peso”.

Além de observar a significância, foi calculado o Coeficiente Phi, sendo encontrado um valor de 0,859 (p < 0,001), indicando uma associação forte. O Kappa não ponderado foi de 0,637 (p < 0,001; IC95%: 0,619–0,654). Contudo, considerando que se trata de uma variável ordinal, o Kappa ponderado pode expressar melhor a concordância, tendo sido encontrado um valor de 0,664 (p < 0,001; IC95%: 0,648–0,681), indicando uma boa concordância para as categorias do estado nutricional. Quando avaliado o Kappa ponderado de forma estratificada (Tabelas 1 e 2), vimos que há boa confiabilidade tanto para sexo (0,681 para homens e 0,646 para mulheres), quanto para idade (0,684 para não idosos e 0,587 para idosos).

**Tabela 1:** Análise bivariada por sexo, seguindo as categorias do índice de massa corporal aferido e do índice de massa corporal referido. Brasil, 2023.

Categoria	Classificação do IMC referido	Classificação do IMC aferido			Total	Kappa ponderado	IC95%	Valor de p
	
Sexo		Baixo peso	Eutrofia	Excesso de peso				
Homens	Baixo peso	104	72	6	182	0,681	0,658–0,704	0,001
	Eutrofia	79	1.067	239	1.385			
	Excesso de peso	11	207	1.468	1.686			
	Total	194	1.346	1.713	3.253			
Mulheres	Baixo peso	103	66	11	180	0,646	0,622–0,671	0,001
	Eutrofia	56	901	346	1.303			
	Excesso de peso	8	158	1.479	1.645			
	Total	167	1.125	1.836	3.128			
Total	Baixo peso	207	138	17	362			
	Eutrofia	135	1.968	585	2.688			
	Excesso de peso	19	365	2.947	3.331			
	Total	361	2.471	3.549	6.381			

IMC: índice de massa corporal; IC: intervalo de confiança.

Fonte: Análise dos autores sobre dados da Pesquisa Nacional de Saúde (PNS) 2019

**Tabela 2:** Análise bivariada por idade, seguindo as categorias do índice de massa corporal aferido e do índice de massa corporal referido. Brasil, 2023.

Categoria	Classificação do IMC referido	Classificação do IMC aferido			Total	Kappa ponderado	IC95%	Valor de p
	
Idade		Baixo peso	Eutrofia	Excesso de peso				
Não idoso	Baixo peso	28	19	2	49	0,684	0,664–0,704	0,001
	Eutrofia	18	678	228	924			
	Excesso de peso	1	108	1.204	1.313			
	Total	47	805	1.434	2.286			
Idoso	Baixo peso	75	47	9	131	0,587	0,555–0,620	0,001
	Eutrofia	38	223	118	379			
	Excesso de peso	7	50	275	332			
	Total	120	320	402	842			
Total	Baixo peso	103	66	11	180			
	Eutrofia	56	901	346	1.303			
	Excesso de peso	8	158	1.479	1.645			
	Total	167	1.125	1.836	3.128			

IMC: índice de massa corporal; IC: intervalo de confiança.

Fonte: Análise dos autores sobre dados da Pesquisa Nacional de Saúde (PNS) 2019

### Análise do IMC Categorizado – Validade de Critério (Acurácia)

Também foram calculadas estatísticas relativas à acurácia, considerando o IMC aferido como padrão-ouro. Para isso, considerou-se como desfecho a ocorrência de excesso de peso. Além disso, foram calculados os parâmetros na forma estratificada para sexo (homens e mulheres) e idade (não idosos e idosos). Os resultados encontrados estão dispostos na Tabela 3.

**Tabela 3:** Parâmetros encontrados para a análise de acurácia entre do índice de massa corporal aferido e do índice de massa corporal referido de acordo com os subgrupos estudados e para a amostra total. Brasil, 2023.

Variável	Sensibilidade	Especificidade	VPP	VPN	Precisão do diagnóstico
				
	% (IC95%)	% (IC95%)	% (IC95%)	% (IC95%)	% (IC95%)
Homens	85,7 (84,0–87,3)	85,8 (84,0–87,5)	87,1 (85,4–88,6)	84,4 (82,5–86,1)	85,8 (84,5–86,9)
Mulheres	80,6 (78,7–82,3)	87,2 (85,2–88,9)	89,9 (88,4–91,3)	75,9 (73,7–78,0)	83,3 (81,9–84,6)
Não idoso	85,8 (84,5–87,0)	85,0 (83,3–86,6)	89,7 (88,5–90,8)	79,6 (77,8–81,4)	85,5 (84,4–86,5)
Idoso	72,1 (68,7–75,3)	89,2 (87,1–91,0)	82,9 (79,8–85,7)	81,4 (79,0–83,7)	82,0 (80,1–83,8)
Amostra total	83,0 (81,8–84,2)	86,4 (85,1–87,6)	88,5 (87,3–89,5)	80,3 (78,8–81,6)	84,6 (83,6–85,4)

VPP: valor preditivo positivo; VPN: valor preditivo negativo; IC: intervalo de confiança.

Fonte: Análise dos autores sobre dados da Pesquisa Nacional de Saúde (PNS) 2019

A sensibilidade, capacidade do indivíduo com excesso de peso de referir seus dados de peso e altura corretamente, foi elevada, 83,0% (IC95% entre 81,8% e 84,2%), enquanto a especificidade, capacidade do indivíduo sem excesso de peso de referir seus dados corretamente, também foi elevada, 86,4% (IC95% entre 85,1% e 87,7%). Quando estratificados por sexo e idade, vemos que mulheres e idosos (≥ 60 anos) com excesso de peso têm menor sensibilidade para a autorreferência de seu estado nutricional, 80,6 e 72,1%, respectivamente.

### Análise do IMC Categorizado – Validade de Constructo

Finalmente, o IMC categorizado foi analisado em função de algumas variáveis preditoras que comumente estão associadas ao estado nutricional, tais como: sexo, raça/cor, escolaridade e renda^8,18^. A análise de regressão de Poisson para IMC referido e para IMC aferido (Tabela 4) mostrou que a única variável que tem diferença nos dois modelos é o sexo (que foi significativo para o aferido e não para o referido). Isso reforça a necessidade de, ao se utilizar o IMC referido, sempre estratificar por sexo, corroborando os resultados anteriores.

**Tabela 4:** Análise por Regressão de Poisson para o índice de massa corporal referido e índice de massa corporal aferido. Brasil, 2023.

IMC	Variável	RP ajustada	IC95%	valor de p
IMC referido	Sexo	1,022	0,974–1,072	0,367
	Cor/Raça	1,031	0,982–1,083	0,217
	Escolaridade	0,781	0,715–0,854	0,001
	Renda familiar	0,94	0,895–0,988	0,014
IMC aferido	Sexo	1,122	1,073–1,174	0,001
	Cor/Raça	1,002	0,958–1,048	0,925
	Escolaridade	0,812	0,748–0,881	0,001
	Renda familiar	0,948	0,906–0,993	0,022

IMC: índice de massa corporal; RP: razão de prevalência; IC: intervalo de confiança.

Fonte: Análise dos autores sobre dados da Pesquisa Nacional de Saúde (PNS) 2019.

## DISCUSSÃO

Por meio da série de análises estatísticas realizadas para validação dos dados de peso e altura aferidos e referidos (e consequente estimativa de IMC), este estudo demonstrou que a técnica de autorreferência pode ser utilizada em inquéritos populacionais, sendo uma estratégia eficiente a fim de minimizar as dificuldades frequentemente encontradas (de logística e tempo, especialmente) em aferir diretamente estas medidas antropométricas.

Houve uma maior reprodutibilidade entre adultos não idosos (18 a 59 anos) quando comparados às pessoas idosas e entre homens quando comparados às mulheres. Essa conclusão é reforçada quando vemos que, dentre as variáveis socioeconômicas e demográficas analisadas, a única que tem diferença em todos os modelos estatísticos é o sexo, corroborando com alguns estudos semelhantes^8,18^.

A sensibilidade entre as mulheres foi de 80,6%, enquanto entre os homens foi de 87,0%, ou seja, entre mulheres e homens com excesso de peso, as mulheres conseguem relatar com menor precisão os seus dados. Em contrapartida, quando sem excesso de peso, as mulheres apresentaram uma especificidade (87,2%) maior, em acordo com outros estudos, que mostraram que aquelas pessoas sem excesso de peso conseguem relatar com maior precisão sobre suas medidas^7^.

O resultado do presente estudo sobre as mulheres reforça a hipótese de que elas, ao mesmo tempo em que apresentam maior preocupação em monitorar suas medidas e estado de saúde^21^, de forma geral, quando comparada aos homens, também têm uma maior tendência a subestimar seu peso e superestimar sua estatura^6,20,22^, o que pode gerar importantes flutuações na definição do seu estado nutricional. Isso pode ser explicado pelo fato desse grupo comumente apresentarem maiores índices de insatisfação corporal, desencadeada pela alta pressão social para atingir determinados padrões de beleza^23,24^. De forma geral, quando os indivíduos se sentem mais satisfeitos com sua própria imagem corporal ou estão mais próximos do peso corporal de seus pares, são menos propensos a informar incorretamente seu peso^21^.

Os idosos apresentaram menor sensibilidade (72,1%) quando comparados aos adultos não idosos (85,0%), ou seja, idosos com excesso de peso conseguem ter uma menor precisão no relato de seus dados antropométricos, ao passo em que idosos sem excesso de peso (especificidade de 89,2%) conseguem realizar esse relato com maior precisão, o que corrobora outros estudos semelhantes^20,25^. Isso pode estar associado ao próprio processo fisiológico do envelhecimento^26^, que leva a uma diminuição de sua estatura ao longo dos anos, bem como uma progressiva redução de massa muscular e/ou aumento de tecido adiposo pela diminuição do ritmo metabólico, impactando em mudanças no peso corporal, muitas vezes não percebidas pela pessoa idosa^27,28^.

Além disso, este é um grupo que, geralmente, não verifica seu peso e altura com regularidade, levando, portanto, a um relato de informações mais imprecisas e desatualizadas, que também pode estar associado ao viés de memória, presente comumente nesse público, corroborando estes resultados com outros estudos que observaram uma subestimação no peso e superestimação da altura em idosos^18,20^.

Esta validação da PNS é muito importante, tendo em vista a larga utilização deste banco de dados em estudos transversais e longitudinais a fim de avaliar a morbidade, o funcionamento da assistência de saúde e o estilo de vida da população brasileira, em diferentes áreas de conhecimento pela riqueza de variáveis, grande tamanho amostral e representatividade de todas as unidades federativas do Brasil.

Ademais, ainda que tenhamos encontrado boa concordância para as categorias do estado nutricional, mostrando que a PNS 2019 pode ser utilizada para estimar prevalência de excesso de peso nesta população avaliada, outros estudos de base populacional diferentes dessa, encontraram valores de subestimação^29,30^ e superestimação quanto ao sobrepeso e obesidade^18^.

Para a validação de constructo foram utilizados alguns indicadores preditores que a literatura comumente mostra associação ao estado nutricional (sexo, raça/cor, escolaridade e renda), o que reforçou a legitimidade da utilização dos dados autorreferidos em estudos de associações, pela sua similaridade com dados aferidos. Portanto, embora seja recomendável cautela ao utilizar o autorrelato para estudos de prevalência do estado nutricional para excesso de peso, especialmente com idosos e mulheres, os resultados do presente estudo indicam que há uma possibilidade concreta de realizar estudos de associação tendo como variável de desfecho o estado nutricional referido.

Isso se torna mais relevante quando se trata de grandes inquéritos populacionais, em que há uma grande demanda de custo e logística para o trabalho de campo, a partir da necessidade de treinamentos, aquisição de materiais adequados e em grandes quantidades, dificuldades com transporte e longa duração das coletas. A utilização de dados antropométricos autorreferidos aumenta a viabilidade dos estudos observacionais, apontando para a importância de um bom planejamento do delineamento da pesquisa, levando em consideração seu objetivo central e as características da população-alvo, especialmente socioculturais, demográficas e econômicas.
